# Outcomes of simultaneous high tibial osteotomy and anterior cruciate ligament reconstruction in anterior cruciate ligament deficient knee with osteoarthritis

**DOI:** 10.1186/s12891-018-2161-0

**Published:** 2018-07-18

**Authors:** Cheng Jin, Eun-Kyoo Song, Quan-He Jin, Nam-Hun Lee, Jong-Keun Seon

**Affiliations:** 1 Department of Orthopedic Surgery, Chinese People’s Armed Police Force, Zhejiang Corps Hospital, Jiaxing, 314000 China; 2 Center for Joint Disease, Chonnam National University Hwasun Hospital, Hwasun, South Korea

**Keywords:** Knee, High tibial osteotomy, Anterior cruciate ligament reconstruction

## Abstract

**Background:**

We aimed to evaluate clinical and radiological results after simultaneous open-wedge high tibial osteotomy (HTO) and anterior cruciate ligament (ACL) reconstruction in patients with ACL deficiency combined with medial uni-compartmental osteoarthritis (OA) and varus deformity.

**Methods:**

This retrospective study was performed using data collected from 2005 to 2011 on a total of 24 patients who were diagnosed with ACL injury and medial unicompartmental OA with varus deformity, and who subsequently underwent simultaneous open-wedge HTO and arthroscopic ACL reconstruction. The mean follow-up duration was 5.2 years. For clinical outcomes, we evaluated Lysholm score, Tegner activity score, range of motion, Lachmann test, and pivot-shift test, and for radiological outcomes, we evaluated the degree of varus deformity, progression of medial OA, tibial posterior slope, anterior instability, and postoperative complication.

**Results:**

There were no limitations in range of motion found in any cases. Three patients showed progressive osteoarthritis on the medial compartment. The mechanical femorotibial angle was significantly corrected from varus 7.0 degrees to valgus 1.2 degrees, and the tibial posterior slope was not significantly changed. The Lysholm and Tegner activity scores were significantly improved after surgery (from 58 to 94 points on the Lysholm scale and from 4.0 to 5.3 points on the Tegner activity scale). Although the Lachman test and the pivot-shift test showed significant improvements after surgery, instability greater than Gr II was observed in three patients on the Lachman test and in four patients on the pivot-shift test. The side-to-side difference improved from 9.6 mm to 4.2 mm postoperatively as assessed using a Telos® arthrometer. There were no cases of nonunion or fixation loss.

**Conclusions:**

Simultaneous open-wedge HTO and ACL reconstruction in patients with ACL injury with medial compartmental OA showed satisfactory functional outcomes and postoperative activity level scores. However, some patients showed residual instability and progression of OA.

**Electronic supplementary material:**

The online version of this article (10.1186/s12891-018-2161-0) contains supplementary material, which is available to authorized users.

## Background

Anterior laxity in anterior cruciate ligament (ACL) deficient knees is a precursor to degenerative osteoarthritis (OA) [1, 2]. The risk of developing radiologically evident OA of at least Kellgren- Lawrence grade 2 is reported to be five-fold higher in the setting of anterior laxity than in uninjured knees [1, 3]. For patients with concomitant varus deformity, ACL reconstruction may restore the stability of the knee, but it cannot prevent the progression of medial compartmental OA [4, 5]. Furthermore, varus alignment adds an adduction moment while patients are walking [6]. This adduction moment force translates to varus thrust on the knee joint during the heel strike [6, 7]. This may stress the reconstructed ACL graft, causing early failure. The success of valgus high tibial osteotomy (HTO) in treating unicompartmental OA in young adults encouraged the authors to attempt the procedure in knees with ACL injuries in order to restore stability and prevent the progression of OA [8–10]. Such a procedure should theoretically unload the medial compartment and decrease the stress on the newly reconstructed ACL graft. However, such a procedure may also increase the risk of morbidity and delay the rehabilitation from the ACL reconstruction. Further, valgus HTO is known to increase the posterior slope of the proximal tibia, which may further accentuate the stress on the reconstructed graft [11, 12]. The indications and benefits of this combined surgery therefore remain unclear in the literature. Variability in pathology, the follow-up period, and clinical scores used for evaluation makes comparisons between the available data difficult [13].

The aim of our study was to evaluate radiological and functional outcomes in patients with ACL deficient knees and medial unicompartmental OA undergoing simultaneous ACL reconstruction and HTO.

## Methods

The institutional review board (Chonnam National University Hospital) approved this retrospective study prior to the commencement of this study. We reviewed the records of all patients who underwent simultaneous ACL reconstruction and HTO from 2005 to 2011 and got the written consents from the patients. The inclusion criterion for the study was an ACL deficient knee with varus deformity (more than 5 degrees) on full-leg length standing radiography as well as radiographically-proven (X-ray or MRI) isolated medial unicompartmental osteoarthritis (≥ Kellgren-Lawrence Gr I) with a minimum follow-up period of 3 years. Other ligamentous laxities, degenerative arthritis of the lateral compartment, tears of the lateral meniscus, inflammatory knee arthritis, and a follow-up period shorter than 3 years were regarded as exclusion criteria.

Two senior authors operated on all patients. Diagnostic arthroscopies were carried out prior to ACL reconstruction and osteotomy in all cases. Cartilage defects on the medial side were dealt with using micro-fractures (eight cases) when it is necessary. Partial meniscectomies (nine cases) or meniscal repair (five cases) were performed whenever deemed necessary by the surgeon. Quadrupled hamstring autografts were harvested from the ipsilateral side in all cases. After the drilling of a femoral tunnel through the outside-in technique using an ACL guide (Linvatec, Largo, FL, USA), medial opening biplanar high tibial osteotomy was performed as described in the literature [14]. The starting point of osteotomy on the medial side was shifted more distally than typically described to allow enough space for the tibial tunnel. The osteotomy was opened to the point at which the mechanical axis passed through the 62.5% point of the tibial plateau. This was fixed with two Aescula plates (Medysey, Seoul, Korea), non-locking plates with rectangular metal wedges. To maintain the tibial slope, the anterior plate was smaller than the posterior plate. A cancellous bone chip allograft was performed when the tibial opening was larger than 10 mm. This was followed by the drilling of a tibial tunnel. The graft was then passed through both the femoral and tibial tunnels and secured using a closed loop Endobutton (Smith & Nephew, Andover, MA, USA) or an adjustable loop TightRope (Arthrex, Naples, FL) on the femoral side and an interference screw on the tibial side. The tibial attachment was further secured using a spiked washer and a cortical screw. Range of motion exercises were begun as soon as possible. Knees were kept in a range of motion brace for 4 weeks. Toe touch partial weight bearing was allowed for the first 4 weeks after surgery, after which progressive tolerable weight bearing was encouraged.

An independent orthopedic fellow who was not directly involved in patient management collected all data. Preoperative and postoperative radiological and clinical records were evaluated. Range of motion, Lyslohm scores, Tegner activity scores, pivot-shift test, and Lachman tests were estimated in the clinical records. A Telos® device (Austin & Associates, Fallston, MD, US) was used to evaluate stress tests. Anterior laxities were evaluated in 30-degree flexion with 15Lb anterior stresses using the Telos device by side to side difference compared with a normal contralateral knee. The anterior-posterior and lateral radiographs were screened for the progression of osteoarthritis and for changes in posterior slope. Lastly, full-leg length standing radiographs taken preoperatively and 3 months postoperatively were assessed for preoperative and postoperative lower limb alignment.

### Statistical analysis

All variables are expressed as means +/− standard deviations. The difference between preoperative and postoperative values was assessed using a paired-t test for continuous variables and a Chi-square test for categorical variables. A *p* value less than .05 was considered to be significant. SPSS software version 22 (IBM, Armonk, NY, USA) was used for all analysis.

## Results

Our records yielded 24 patients that satisfied the inclusion criterion, consisting of 20 males and four females. The average age of our patients was 40.2 years (range, 29–52 years). The mean follow-up period was 5.2 years (range, 3–9 years). None of the patients required conversion to arthroplasty.

Table 1 presents the preoperative and final postoperative clinical and radiological outcomes of our study. At the final follow up, none of the patients had any range of motion (ROM) limitations. Clinical scores improved in all cases to pre-injured levels. The mechanical axis was corrected from preoperative varus 7.0 to a mean of valgus 1.2 degrees. None of the patients showed significant changes (more than three degrees from preoperative values) in posterior slope.

**Table 1:** Preoperative and postoperative clinical and radiological results

	Preoperative	Final follow-up	*P*-value
Mechanical axis (in degree)	7.0 ± 2.3	−1.2 ± 1.4	< 0.001
Tegner activity score	4.0 ± 1.1	5.3 ± 0.9	< 0.001
Lysholm score	58.5 ± 12.0	94.0 ± 5.9	< 0.001
Anterior laxity on Telos stress radiography (in mm)	9.6 ± 2.8	4.2 ± 2.6	< 0.001
Posterior slope	9.1 ± 1.4	10.2 ± 2.3	0.092
Range of motion	137.3 ± 6.2	136.5 ± 6.0	0.691

-; valgus

Clinically, anterior laxity was improved in all patients except for three who showed grade 2 laxity on the Lachman test and four who had a positive pivot-shift test (Table 2). Stress radiography confirmed the clinical findings of improved laxity, with a significant decrease in side-to-side difference in anterior laxity compared to preoperative values.

**Table 2:** Improvement of anterior instability based on Lachman and pivot shift test

Grade	Lachman test		P-value	Pivot-shift test		P-value
	Preoperative	Final follow-up		Preoperative	Final follow-up	
0	0	14	< 0.001	0	15	< 0.001
I	3	7		9	5	
II	14	3		11	4	
III	7	0		4	0	

**Fig. 1:**
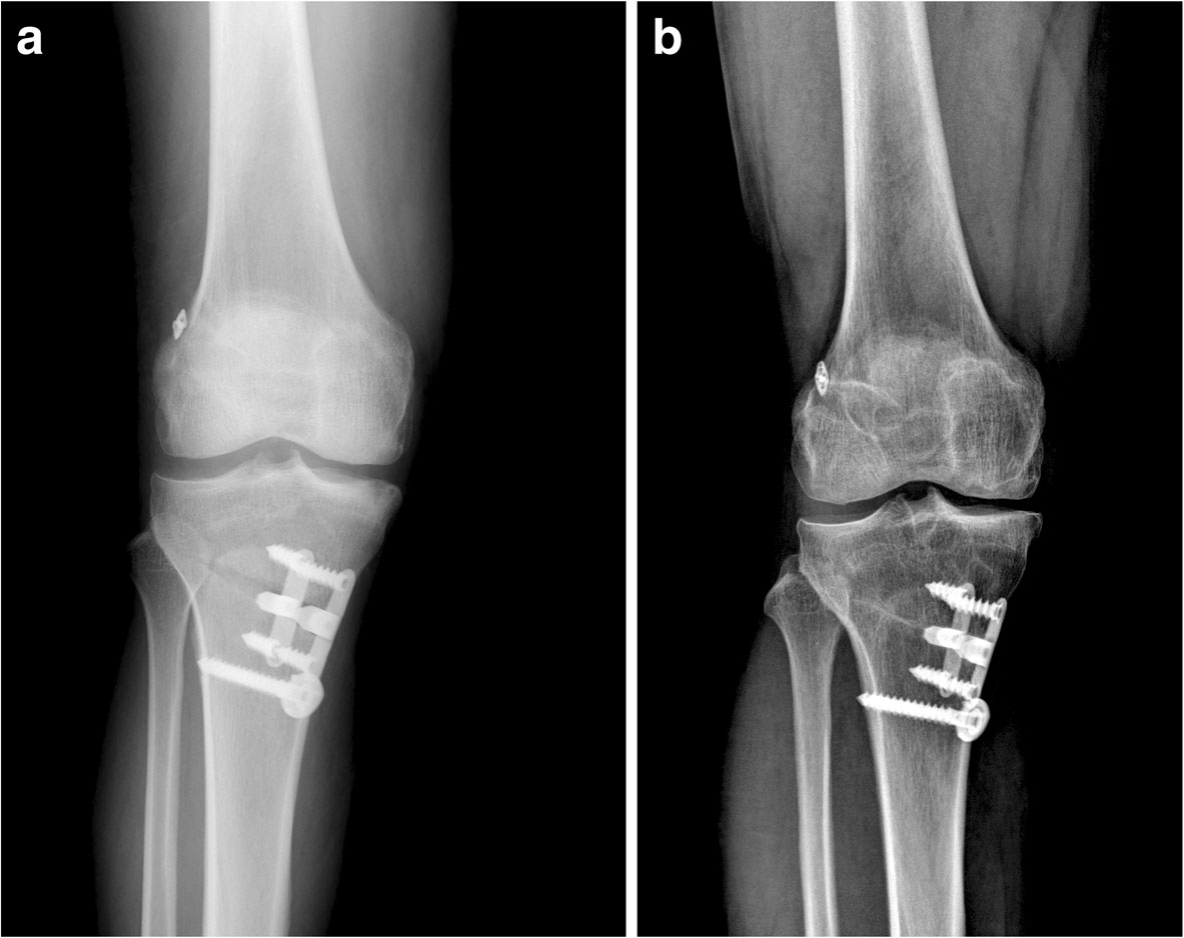
**a** Immediate operative knee x-ray showing medial compartment osteoarthritis following ACL reconstruction and valgus high tibial osteotomy. **b** Knee x-ray taken three years following ACL reconstruction and valgus high tibial osteotomy that shows the progression of arthritis of medial compartment

At the final follow-up, three patients showed progression of medial unicompartmental osteoarthritis (Table 3, Fig. 1). Two of them had medial meniscal damage and one had full thickness cartilage defect on medial femoral chondyle at the time of index surgery.

**Table 3:** Progression of osteoarthritis on medial compartment

Grade	Preoperative	Final follow-up	P-value
I	10	8	0.682
II	9	10	
III	5	6	
IV	0	0

Three patients complained of hyperesthesia in the antero-lateral part of the proximal tibia and one patient had pain in the incision site. There was no case of nonunion of the osteotomy site. No other major complications were noted in our series.

## Discussion

The most important finding of our study was that simultaneous ACL reconstruction and valgus HTO showed relatively good clinical and radiological outcomes in ACL deficient knees with osteoarthritis. However, some patients showed a moderate degree of instability or a progression of OA in spite of ACL reconstruction. Further studies are warranted to clearly define a patient subgroup that would benefit from the combined procedure.

Patients with combined laxity and medial compartmental OA tend to be young and therefore retaining their natural knee is a priority [15]. With the aim of restoring the laxity and alignment of the knee, many authors have suggested a combined ACL reconstruction and valgus osteotomy procedure [8–10]. Consistent with the results previously published by most authors, we found that none of our cases required conversion to total knee arthroplasty over a mid-term follow-up of an average of 5 years [8–10, 12, 16]. This substantiates the role of the combined procedure in such knees.

Most previous authors reported improved functional scores following combined surgery. We had a similar experience as we found Lysholm scores and Tegner activity scores to be significantly better after the operation than they were before the operation [8–10, 16–18]. All patients subjectively reported better stability of the knee. Despite these encouraging results, six patients (25%) showed progressive medial compartmental osteoarthritis on serial radiographs. Similarly, four patients (17%) had a positive pivot shift test, three of whom also had a positive grade 2 Lachman test. Complication rates in the literature are variably defined. In line with our experience, Zaffanigni et al. [19] reported 22% progression in medial compartmental OA after a 6.5-year follow-up. In contrast, Dejour et al. [20] found no difference in the OA rate preoperatively vs. postoperatively in a retrospective study.

A similar discrepancy exists in the rate of anterior laxity. Lattermann reported that 31% of patients developed graft insufficiency [8]. Consistent with our results, Schuster et al. [21] reported graft insufficiency in 18% of patients. However, others have described a 0% rate of graft insufficiency following the combined procedure [22]. Finally, contrary to our results, Dean et al. [23] conducted a systemic review and identified knee stiffness as the most common complication. We observed no loss of movement in any patient. These differences between reports may be the result of the use of different surgical techniques as well as differences in preoperative patient conditions, timings of the surgeries, rehabilitation protocols, and/or follow-up periods. The published data is heterogeneous regarding each of the above parameters, making comparisons difficult. Such wide differences in complication rates highlight the currently inadequate understanding of the true pathological nature of ACL deficiency with unicompartmental OA. It further stresses the need for future studies to define indications for the combined procedure. In addition, the combined procedure is a complex technique that requires meticulous preoperative planning, skilled intraoperative techniques, and an aggressive postoperative rehabilitation protocol.

Changes in tibial slope following high tibial osteotomy are well known to occur and may affect the stresses on a reconstructed graft [11]. We observed no significant differences between preoperative and postoperative values. However, despite maintenance of the slope in all cases, we observed graft insufficiency in 17% of cases, indicating that a non-mechanical mechanism may be involved in preventing graft healing. Consistent with our experience, Schuster et al. hypothesized that severe osteoarthritic changes and an associated catabolic intra-articular milieu may play a role in insufficient graft integration or compromise graft functionality [18].

There are a few limitations to our study. First, it is a retrospective study and is therefore subject to the associated usual biases. Secondly, the sample size is too small to draw any definitive conclusions about the outcomes of this combined procedure. Thirdly, we carried out abrasion chondroplasty in the same setting, and as its role on ACL healing is still debatable, it may act as a confounding factor. The last limitation was that the majority of our patients were male and only four patients were female. Despite these limitations, our study strongly indicates a need for caution when choosing patients and performing combined ACL reconstruction and valgus HTO surgery, because of the possibility of a high complication rate.

## Conclusions

Simultaneous open wedge HTO and ACL reconstruction showed satisfactory correction angles and improved knee joint function, delaying the need for arthroplasty. However, this combined procedure should be undertaken with caution, as it is associated with a high rate of laxity and the progression of osteoarthritis.

## Additional file

Additional file 1: Clinical outcomes including stability and radiologic results of patients. (XLSX 14 kb)

## Abbreviations

ACL: Anterior cruciate ligament
HTO: High tibial osteotomy
OA: Osteoarthritis
ROM: Range of motion
